# A Case Report on Charcot-Marie-Tooth Disease with a Novel Periaxin Gene Mutation

**DOI:** 10.7759/cureus.5111

**Published:** 2019-07-09

**Authors:** Sorabh Datta, Saurabh Kataria, Raghav Govindarajan

**Affiliations:** 1 Neurology, University of Missouri, Columbia, USA

**Keywords:** neurology, sensorimotor neuropathy, congenital, gene expression, genetic mutation, protein, pes cavus, demyelinating diseases, charcot-marie-tooth, autosomal recessive disorder

## Abstract

Charcot-Marie-Tooth (CMT) disease is one of the most common primary hereditary neuropathies causing peripheral neuropathies. More than 60 different gene mutations are causing this disease. The *PRX* gene codes for Periaxin proteins that are expressed by Schwann cells and are necessary for the formation and maintenance of myelination of peripheral nerves. Dejerine-Sottas neuropathy and Charcot-Marie-Tooth type 4F (CMT4F) are the two different clinical phenotypes observed in association with *PRX* gene mutation. This article describes a case of an elderly male with a novel mutation involving the *PRX* gene.

## Introduction

As per the Dyck classification in the year 1970, primary hereditary neuropathies are divided into hereditary motor sensory neuropathy (HMSN) and hereditary sensory autonomic neuropathy (HSAN) [1]. Charcot-Marie-Tooth (CMT) disease is a type of HMSN with an estimated prevalence of 1 in 2,500 [2]. CMT can follow autosomal recessive (ARCMT), X-linked recessive, and also an autosomal dominant pattern. CMT type 4 is a rapidly increasing ARCMT disease form in HMSN, although CMT type 1 and 2 still account for the most substantial proportion of the patient population [3]. CMT4F is a severe, demyelinating subtype of CMT type 4 and is characterized by childhood onset of slowly progressing weakness in the distal muscles associated with atrophy. It also presents with pes cavus and hammer-toe deformities. It was first reported in a consanguineous Lebanese family by Guilbot A, et al. in the year 2001 [4]. This particular subtype involves *PRX* gene mutation. The normal function of the *PRX* gene is to encode L- and S-periaxin proteins. L- and S-periaxin protein is vital in the Schwann cell myelination process and mostly localized at the abaxonal membrane where it helps in stabilizing the myelin sheath [5-6]. The theory of L- and S-periaxin protein in stabilizing the myelin sheath can further be supported by the study carried out by Gillespie CS, et al. in the year 2000, where the mice with null *PRX* gene mutation showed substantial peripheral nervous system (PNS) demyelination [7]. Sural nerve biopsy gives a good immunochemistry sample for gene mutation diagnosis [5]. Mutations in the *PRX* gene lead to early-onset demyelinating neuropathies, namely CMT4F and Dejerine-Sottas neuropathy [4-5]. Herein, we report a case with an early-onset demyelinating disease caused by a novel *PRX* gene variant.

## Case presentation

A 71-year-old male patient with a past medical history of hypertension and coronary artery bypass surgery (CABG) presented to the neurology clinic with weakness in both lower extremities, progressively worsening tingling and numbness, and difficulty in getting up from the chair. He had a history of having laxity in both ankles since his 20s. He also had a tendency of his feet to turn inwards, trip, and subsequently sprain his ankles. Also, the patient reported that his balance and gait had worsened in the last few years leading him to use orthotics for one to two years. The patient denied any significant alcohol intake. Family history also reported having hammer toes in immediate family members involving his mother, sibling, and daughter.

Physical exam was remarkable for bilateral high arched feet, pes cavus deformity with Achilles contracture, wasting of distal legs and feet, and weakness in bilateral toe flexors and scoliosis. Sensations were decreased to pinprick bilaterally just below the knee in the lower limbs and at the wrist crease in the upper limbs. Proprioception was impaired in toes (great toe) and fingers (index finger) bilaterally. The vibration was absent at the feet, ankle, and the hand. Hip flexion and knee flexion were 4/5 bilaterally. Foot dorsiflexion was 3/5 bilaterally and foot plantar flexion was 4/5 bilaterally. The patient could not stand on his heels and could not do tandem gait. Romberg’s sign was positive.

The initial lab studies were remarkable for mildly elevated creatinine phosphokinase (CPK) at 295 U/L, normal complete blood count (CBC), complete metabolic profile, HbA1C, Vitamin B12, serum protein electrophoresis (SPEP), kappa/lambda ratio, and TSH levels. Antidromic sensory nerve conduction study (NCS) of bilateral sural nerve showed reduced sensory nerve action potential amplitude (right = 0.5 uV and left = 0.6 uV, normal >5 uV). NCS of the right peroneal motor nerve showed significantly reduced compound muscle action potential amplitude (0.6 mV), and NCS of the right tibial nerve was absent. Right median motor NCS showed reduced CMAP amplitude (4 mV, normal >5 mV) with slowed conduction velocity of 25 m/s (normal >40 m/s). Hereditary neuropathy panel through GeneDx was sent, which reported the presence of a novel heterozygous mutation of the *PRX* gene with K1062N (p.Lys1062Asn). 

## Discussion

The Periaxin protein is encoded by the gene Periaxin (*PRX*), which is located on chromosome 19 [8]. The Periaxin proteins (L and S) are the key molecules that are necessary for the maintenance of peripheral nerve myelination and Schwann cell compartmentalization [9]. L-periaxin protein is involved in connecting Schwann cells to the basal lamina (abaxonal membrane) by interacting with the dystroglycan complex (DGC) through dystrophin-related protein type two (DRP2) [6,9-10]. This interaction is necessary for the formation of cytoplasmic compartments called Cajal bands that are associated with proper elongation of Schwann cells during development and internodal length [11]. *PRX* gene mutation causes dysfunction in the protein function mainly due to a truncated protein and loss of the acidic domain which is hypothesized to prevent L-periaxin from binding to the cytoskeleton of the Schwann cells and thereby hindering the production of stable myelin. This is likely the pathogenesis involved in *PRX*-related neuropathies [9,12-13].

*PRX* mutations cause early-onset, autosomal recessive demyelinating CMT4F, and Dejerine-Sottas disease; their clinical phenotypes are severe [4-5]. The classic type of CMT4F neuropathy presents with early-onset symptoms at age less than seven years, sensory neuropathy that shows little to no progression over time, sensory loss and/or sensory ataxia, slow nerve conduction velocity, distal muscle weakness, pes cavus, delayed walking, scoliosis and absent deep tendon reflexes [9]. Phenotypic variability also has been reported, and a subset of patients with a milder phenotype, characterized by adult-onset sensory neuropathy, vocal cord paralysis, and scoliosis, has been described by Tokunaga, et al. [8].

In the *PRX* gene mutation analysis, we performed a sequencing and deletion/duplication analysis of 53 genes included in this panel. The results were coding DNA c.3186 G >T, semi-conservative amino acid substitution lysine 1062 Asparagine (K1062N) variant. The novel K1062N mutation is a type of nonsynonymous substitution with semi-conservative amino acid replacement. This substitution that may have an impact on the secondary structure of the protein, as these residues differ in some properties suggesting that it might be a part of an important functional domain in *PRX*. More research is needed to clarify which gene mutation is the most common in adult-onset CMT disease. 

Our investigation, in this case, will help in affirming the importance of the specific proteins in peripheral nerve function and also describing the clinicopathological results of their dysfunction.

## Conclusions

CMT4F is an autosomal recessive demyelinating motor sensory neuropathy involving a mutation in the *PRX* gene. The severe phenotype is characterized by childhood onset of slowly progressing weakness in the distal muscles associated with atrophy. It also presents with pes cavus and hammertoe deformities, while we have described herein the milder phenotype case of CMT4F that presents in adulthood. Further research is needed to have a better understanding of the *PRX* gene mutation and its phenotypes.
